# The catch 22 of condoms in US correctional facilities

**DOI:** 10.1186/1471-2458-7-296

**Published:** 2007-10-21

**Authors:** Joseph D Tucker, Suzanne W Chang, Jacqueline P Tulsky

**Affiliations:** 1 Division of Infectious Diseases, Massachusetts General Hospital, Jackson 504, 55 Fruit Street, Boston, MA, 02139, USA; 2 Department of Medicine, University of California San Francisco, San Francisco, CA, USA; 3 Department of Medicine and the Positive Health Program, University of California San Francisco, San Francisco, CA, USA

## Abstract

**Background:**

Despite the high prevalence of sexually transmitted infections (STIs) and HIV infection in US correctional settings, most jails and prisons in the United States prevent inmates from using condoms to prevent STIs/HIV.

**Discussion:**

This article makes the following arguments to justify a scalable and feasible next step in the prevention of HIV/STIs among inmates: condoms are a basic and essential part of HIV/STI prevention, HIV/STI transmission occurs in the context of corrections, and several model programs show the feasibility of condom distribution in prisons. A lower end estimate for HIV incidence among incarcerated applied to 2,000,000 new inmates annually results in thousands of new HIV infections acquired each year in corrections that could be prevented with condoms in corrections facilities. Programs from parts of the United States, Canada, and much of Europe show how programs distributing condoms in correctional facilities can be safe and effective.

**Summary:**

Public health and corrections officials must work together to ensure that condoms and broader sexual disease prevention programs are integrated into US jail and prison health systems.

## Background

"Orr would be crazy to fly more missions and sane if he didn't, but if he was sane he had to fly them. If he flew them he was crazy and didn't have to; but if he didn't want to he was sane and had to."

-*Catch-22*, Joseph Heller

A US prison medical provider would be reasonable to give an inmate a condom to prevent HIV and sexually transmitted infections (STIs) but doing so would acknowledge that unprotected sex and sexual HIV transmission occur in jails and prisons. Current policies in the vast majority of US prisons hold that sex in prison is illegal and condoms are contraband. This is the Catch-22 of condoms in US prisons and jails. The data connecting prisoners to both higher seroprevalence of HIV/STI [1] and HIV seroconversion from sex during incarceration [2] is clear; condoms are similarly well understood to prevent HIV/STIs. Although public health experts and physicians have called for more attention to HIV/STI treatment and prevention in jails and prisons [3], a plan of action and detailed account of successful programs has not emerged. This article makes the following arguments to justify a scalable and feasible next step in the prevention of HIV/STIs among inmates: condoms are a basic and essential part of HIV/STI prevention, HIV/STI transmission occurs in the context of corrections, and several model programs show the feasibility of condom distribution in prisons.

## Discussion

The importance of condoms for sexual HIV prevention among inmates and within correctional settings has been known for some time [4,5]. Condoms are a core component of basic HIV prevention services recommended by the US Centers for Disease Control and the World Health Organization [6,7]. The WHO recommendations on HIV in prisons specifically calls for widespread condom availability for all inmates [8]. The Institute of Medicine has argued for expanded STI services among disadvantaged populations [9]. The Institute recommended that detention facilities provide comprehensive STI-related services, including counseling and education, screening, diagnosis and treatment, partner notification and treatment, as well as methods for reducing unprotected sex.

Several studies highlight that unprotected sex facilitates HIV and STI transmission in correctional settings [10-16]. Seroprevalence data indicate that HIV seroprevalence among incarcerated individuals is fivefold greater than the seroprevalence among the general population [17]. Most HIV positive inmates enter the correctional system with infection, and do not acquire it during incarceration. Lack of testing upon entry or release in prisons obscures the extent to which HIV negative inmates acquire HIV during prison stays. There are currently 19 states with mandatory HIV testing on entry, and Centers for Disease Control data from one state (Georgia) recently investigated HIV seroconversion in correctional settings. In a study of 17 years of HIV testing data, 88 HIV positive individuals who seroconverted during incarceration were identified [2]. Although this corresponds to a low incidence of HIV infection in prisons, the number of individuals diagnosed with new HIV infections in prison settings is heavily influenced by testing policies. The majority of new HIV infections in the Georgia corrections system were discovered during a period when voluntary annual HIV testing was available to inmates. In other studies, even after controlling for the six-month window period between infection and serologic detection, annual HIV transmission rates in prison ranged from 0.3 to 0.63 percent [10-13]. The lower end estimate for HIV incidence among incarcerated (0.3%) among 2,000,000 new inmates annually [17] results in 6,000 new HIV infections acquired each year in corrections that could be prevented with condoms in corrections facilities. Outbreaks of syphilis [14,18], gonorrhea [19], and Hepatitis B[15,16] in prisons provide further support that unprotected sex occurs in jails and prisons. Studies of sexual behaviors in prisons are limited by recall bias and confidentiality, but similarly show high risk behaviors occurring in prisons and jails [4].

Analyzing the critiques of condom distribution in prison is essential to understanding current correctional HIV prevention policy. As the 1990s saw major developments in HIV law outside of the US permitting use of condoms in prisons and jails, concerns about transport of contraband and use of condoms as weapons plagued American correctional facilities. These hesitations about the safety, acceptability, and feasibility of providing condoms to prisoners have been addressed by successful model programs in many US cities and states (San Francisco, District of Columbia, Los Angeles, Philadelphia, parts of NYC, Mississippi, Vermont) [20]. For example, most correctional officers and inmates at the Washington, DC jail, which has provided condoms in jails over ten years, favored condom distribution. The majority of inmates felt there was no increase in sexual activity as a result of condom availability. In addition, the vast majority (87%) of correctional officers reported no problems with this policy [20]. While these US cities and states provide experience to support condom distribution, these programs are dwarfed in breadth and depth by other country's programs.

Large scale national programs making condoms available in prisons have been present in Canada and many European nations for over a decade. The proportion of European prison systems allowing condoms rose from 53% in 1989 to 81% in 1997 [21]. More importantly, none of the penal systems that have introduced condom distribution have reversed their policy, and the number of correctional facilities with condoms grows each year. The Canadian HIV/AIDS Legal Network and the Canadian AIDS Society argued early in the 1990s for more widespread condom availability independent of inmates asking for them [21]. This policy was adopted by the Canadian government, and has proven feasible and effective [22]. Canadian law now guarantees that condoms be available in three discrete unique locations in the prison, in addition to being provided for conjugal visits [23]. In Australia, 50 prisoners brought legal action against the state for non-provision of condoms, prompting the provision of condoms in New South Wales. This policy has since been found effective and sustainable [24]. Stigma associated with obtaining condoms in prison environments did not limit the utility of the program since condoms were available in multiple locations without asking a physician; such measures would be important to ensuring that the stigma associated with homosexual behaviors often found in correctional settings does not limit opportunities for HIV prevention. The increasing number of international jails and prisons distributing condoms provides useful information about structuring scalable successful programs.

## Summary

Basic HIV prevention services only begin with the widespread availability of condoms. While the stigma associated with homosexual behaviors and condom use in prisons would be difficult to change, providing prisoners direct access to condoms could serve to limit the stigma attached to these risk behaviors. Security, medical and public health groups must collaborate to form policy introducing condoms, HIV education, and comprehensive STD screening in jails and prisons. Experiences from several parts of the US, Canada, and much of Europe show that condoms can safely and effectively prevent STIs in prisons. Leverage from lawyers and activists to characterize how prisoners are currently denied their right to the most basic HIV prevention tools may help serve to catalyze change. State and national politicians in the US have identified this as important issue worthy of legislative action. Neither federal [25] nor statewide[26] legislative efforts have successfully resolved the Catch-22 of ensuring condom access among incarcerated individuals in the United States. Public health and corrections officials must work together to ensure that condoms and broader sexual disease prevention programs are integrated into US jail and prison health systems.

## Competing interests

The author(s) declare that they have no competing interests.

## Authors' contributions

JDT conceived of paper, and all authors were involved in writing and editing the manuscript. All authors read and approved the final manuscript.

## Pre-publication history

The pre-publication history for this paper can be accessed here:


